# Seroprevalence of autoimmune antibodies in degenerative ataxias: a broad, disease-controlled screening in 456 subjects

**DOI:** 10.1007/s00415-023-11900-1

**Published:** 2023-07-28

**Authors:** Benjamin Roeben, Madeleine Scharf, Ramona Miske, Bianca Teegen, Andreas Traschütz, Carlo Wilke, Milan Zimmermann, Christian Deuschle, Claudia Schulte, Kathrin Brockmann, Ludger Schöls, Lars Komorowski, Matthis Synofzik

**Affiliations:** 1 Department of Neurodegeneration, Hertie Institute for Clinical Brain Research (HIH), University of Tübingen, 72076 Tübingen, Germany; 2 German Center for Neurodegenerative Diseases (DZNE), 72076 Tübingen, Germany; 3 Institute for Experimental Immunology, affiliated to EUROIMMUN Medizinische Labordiagnostika AG, Lübeck, Germany; 4 Clinical Immunological Laboratory Prof. Dr. Med. Winfried Stöcker, Lübeck, Germany; 5 Division Translational Genomics of Neurodegenerative Diseases, Hertie-Institute for Clinical Brain Research and Center of Neurology, University of Tübingen, Hoppe-Seyler-Str. 3, 72076 Tübingen, Germany

## Dear Sirs,

A growing number of autoimmune antibodies (AA) targeting cerebellar antigens have been associated with autoimmune cerebellar degeneration and/or clinically related ataxia in recent years [1–4]. While classical AAs (e. g. Hu [5], Yo [6], Ri [7], CV2/CRPM5 [8], Amphiphysin [9], Ma2 [10]) are more relevant in paraneoplastic cases, various novel antibodies have been identified to explain hitherto unexplained, sporadic ataxia cases (e. g. ITPR1 [11, 12], neurochondrin [13], Homer-3 [14, 15]). The relevance of AAs in ataxias is strengthened by the recently proposed concept of Latent Autoimmune Cerebellar Ataxia (LACA) highlighting that AAs can occur not only in classical autoimmune ataxia syndromes with (sub-)acute onset, but also in slowly progressive ataxia resembling degenerative cerebellar ataxia (DCA) [16].

Both the growing number of novel AAs and the new LACA concept highlight the need for a comprehensive, well-controlled investigation of the frequency and role of AAs in ataxias with a DCA-disease course. While clinical red flags [17] and diagnostic criteria for Primary Autoimmune Cerebellar Ataxia (PACA) [18] have been proposed, the prevalence of AAs targeting cerebellar antigens and their potential pathophysiological contribution to sporadic and genetic DCAs with their characteristic non-acute, slowly progressive disease course are still to be determined – in particular against strong control cohorts.

We here aimed to evaluate the seroprevalence of AAs leveraging a comprehensive panel of antibodies – including even latest AAs – associated with autoimmune ataxia by a comprehensive screening of several large cohorts of DCAs—including (i) sporadic (i.e. etiologically unsolved) versus (ii) MSA-C and (iii) genetic ataxias (GA) (i.e. both etiologically explained elsewise)—compared to age- and sex-matched groups of (iv) Parkinson’s Disease (PD) patients (as disease controls) and (v) healthy controls (HC).

A total of 228 patients with DCA was consecutively recruited from the ataxia clinic at the Center of Neurology, Tübingen, Germany. The overall DCA cohort comprised three subgroups: patients with (i) etiologically unsolved sporadic adult-onset ataxia (SAOA; n = 71), (ii) clinically probable Multiple System Atrophy cerebellar type (MSA-C; n = 52) according to established clinical diagnostic criteria [19]; or (iii) genetic ataxia (GA; n = 105).

SAOA was defined according to standard criteria [20] as degenerative (i.e. non-acute onset, slowly progressive disease course) ataxia with age at onset ≥ 40 years, negative family history, negative genetic screening for SCA 1, 2, 3, 6, 7 and 17, absence of features indicative of MSA-C and absence of secondary ataxia causes. For an overview of the diagnoses of the GA group, see Supplementary Table 2.

As controls, we assembled age- and sex-matched groups of both healthy (HC; n = 114) and disease controls (PD patients; n = 114).

All patients underwent neurological examination according to a standardized assessment protocol by experienced movement disorder neurologists. Results of routine cerebrospinal fluid analyses were aggregated from medical records as available. For all ataxia patients, severity of ataxia was assessed using the Scale for the Assessment and Rating of Ataxia (SARA) [21]. An individual cross-sectional disease progression rate (CSPR) was calculated by dividing the SARA score at last examination by the disease duration in years.

Serum samples were acquired using standard blood sampling procedures, frozen at −80 °C within 90 min after collection, stored in the local biobank and analysed without any previous thaw-freeze cycle.

Sera of all subjects were screened for a comprehensive panel of 38 antibodies known to target cerebellar antigens or to cause autoimmune ataxia or cerebellar degeneration (see Supplementary table 1) by indirect immunofluorescence assay (IFA) and immunoblot (see Supplementary methods for details). Diagnostic IFA endpoint titres were defined as the highest dilution showing visible fluorescence (see Supplementary table 1).

The study was performed in accordance with the ethical standards as laid down in the 1964 Declaration of Helsinki and its later amendments and was approved by the Ethics Committee of the University of Tuebingen (Nr. 591/2017BO2). All participants gave written informed consent. Data supporting the findings of our study is available upon reasonable request to the corresponding author.

Statistical analyses were performed using IBM SPSS Statistics 28 (IBM, Armonk, NY). Data distribution was assessed both with Shapiro–Wilk tests and Q-Q plots. Normal distributed data were assessed by two-sided t-tests, non-normally distributed data by the Kruskal–Wallis test or the Mann–Whitney-*U* test as appropriate, and nominal variables were assessed by the χ^*2*^ test.

Demographic and clinical characteristics are shown in Table 1. Gender distribution was not different across all groups. Within the DCA group, the GA group was significantly younger, and age of onset significantly earlier, than in the SAOA and MSA-C group (*p* < 0.001); the SAOA group was significantly younger, and age of onset significantly earlier, than in the MSA-C group (*p* = 0.002).

**Table 1:** Demographic and clinical characteristics of the Degenerative Cerebellar Ataxia (DCA), disease control Parkinson’s Disease (PD) and healthy control (HC) group, and comparison of the seropositive versus the seronegative DCA group

	*Degenerative cerebellar ataxias (DCA)*				*PD (n* = *114)*	*HC (n* = *114)*	*p*
	*SAOA (n* = *71)*	*MSA-C (n* = *52)*	*Genetic(n* = *105)*	*Overall (n* = *228)*			
Females, %^a^	28 (39.4)	25 (48.1)	48 (45.7)	101 (44.3)	54 (47.4)	54 (47.4)	ns
Age, years^b,c^	61.0 (15.1)	68.2 (9.6)	50.8 (16.4)	59.0 {23.0}	60.5 {21.0}	63.0 {21.0}	%% | &&& | §§§
Age at onset, years^c^	44.9 (17.9)	58.6 (9.5)	29.5 (18.9)	40.9 (20.4)	NA	NA	%%% | &&& | §§§
Disease duration, years^c^	16.1 (10.2)	10.7 (10.9)	21.5 (12.0)	17.4 (12.0)	NA	NA	%%% | &&& | §§§
SARA score^c^	13.9 (7.2)	19.2 (6.8)	16.9 (8.0)	16.5 (7.7)	NA	NA	%%% | &&
Cross-sectional progression rate^c^	1.1 (0.6)	2.5 (1.6)	1.0 (0.5)	1.3 (1.1)	NA	NA	%%% | §§§
Seropositivity, %^a^	2 (2.8)	0 (0)	3 (2.9)	5 (2.2)	1 (0.9)	0 (0)	ns

*Seropositive versus seronegative DCA group*			
	*Seropositive DCA group* *(n* = *5)*	*Seronegative DCA group* *(n* = *223)*	*p*
Females, %^a^	1 (20.0)	100 (44.8)	0.269
Age, years^c^	53.6 (15.9)	58.0 (16.3)	0.504
Age at onset, years^c^	39.6 (13.5)	40.9 (20.6; MV = 2)	0.714
Disease duration, years^c^	14.0 (5.1)	17.4 (12.1; MV = 1)	0.801
SARA score^c^	7.8 (5.9)	16.7 (7.7; MV = 17)	**0.016**
Cross-sectional progression rate^c^	0.7 (0.5)	1.3 (1.1; MV = 17)	0.092

Significant results are represented with one symbol for *p* < 0.05, two symbols for *p* < 0.01, three symbols for *p* < 0.001 and *ns* for *p* > 0.05. # overall vs PD, $ overall vs HC, % SAOA vs MSA-C, & SAOA vs Genetic, § MSA-C vs Genetic. NA not available, MV missing values. *a* categorical variable, *x*^*2*^ test, absolute values (%). *b* normal distributed continuous variable, Student’s *t*-test, mean (standard deviation). *c* non-normal distributed continuous variable, Kruskal–Wallis test or Mann–Whitney *U* test as appropriate; median (interquartile range)

Disease duration was longest in the GA group (mean disease duration 21.5 ± 12.0 years), followed by the SAOA group (16.1 ± 10.2 years) and the MSA-C group (10.7 ± 10.9 years). SARA score was highest in the MSA-C group (mean SARA score 19.2 ± 6.8 points), followed by GA (13.9 ± 7.2) and the SAOA group (13.9 ± 7.2). Individual CSPR was also highest in the MSA-C group (mean CSPR 2.5 ± 1.6 points/year), followed by the SAOA group (1.1 ± 0.6) and the GA group with the lowest CSPR (1.0 ± 0.5).

**Fig. 1:**
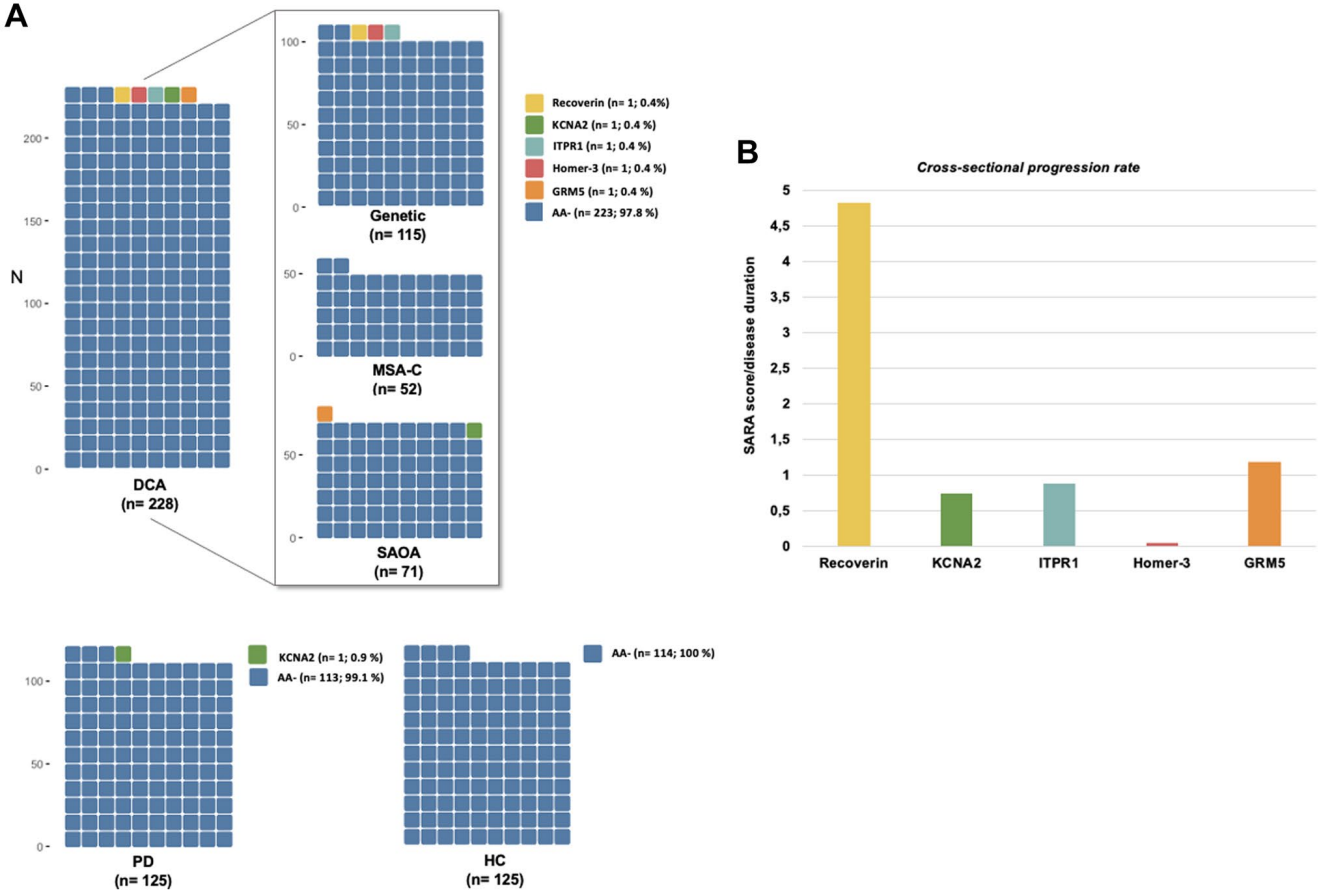
Seroprevalence for autoimmune antibodies (AAs) across DCAs compared to healthy controls and a disease control cohort (PD patients). **A** Seropositivity was a low-prevalent finding in DCAs, not significantly higher than in HC (0%) or PD (0.8%), nor comparing the DCA subgroups (SAOA vs MSA-C vs GA). **B** Age at onset, **C** disease duration and **D** cross-sectional progression rates are shown for the seropositive AAs, respectively. DCA Degenerative Cerebellar Ataxias, PD Parkinson’s disease, Autoimmune Antibody negative group AA-, SAOA Sporadic Adult-Onset Ataxia, MSA-C Multiple System Atrophy cerebellar type, GA Genetic Ataxia

Seropositivity was a low-prevalent finding in DCA patients (5 patients = 2.2%; Fig. 1A), with the following AAs detected: Recoverin (n = 1 GA; titre: 1:32); KCNA2 (n = 1 SAOA; 1:32); Homer-3 (n = 1 GA; 1:1000,); ITPR1 (n = 1 GA; 1:100); GRM5 (n = 1 SAOA; 1:32) (Fig. 1A). KCNA2 antibodies were also detected in one PD patient (titre: 1:320).

Seropositivity in the DCA group was also not significantly higher than in the HC group (0%; p = 0.111) or PD group (0.9%; p = 0.382 (Table 1). Within the DCA cohort, seropositivity was low-prevalent in the SAOA group (2.8%), but also in the GA group (2.9%), while no seropositive case was detected in the MSA-C group (0%). The rate of seropositivity was not statistically significantly different between the three DCA groups (SAOA vs MSA-C: *p* = 0.222; SAOA vs GA: *p* = 0.987; MSA-C vs. GA: *p* = 0.218; Table 1).

The seropositive DCA group did not show higher disease severity or CSPR compared to the seronegative DCA group (Table 1). Routine CSF analyses were available in 5 of the 6 seropositive patients (4 DCA, 1 PD), all yielding unremarkable findings.

On the single subject level, disease progression (CSPR) in patients with seropositivity for KCNA2 (0.75 points/year), Homer-3 (0.05 points/year), ITPR1 (0.89 points/years) and GRM5 (1.20 points/year) (Fig. 1B) was each comparable to the mean CSPR of the seronegative DCA group (1.3 ± 1.1 points/year; Table 1B), while one GA patient with Recoverin positivity showed a remarkably higher CSPR (4.83 points/year; Fig. 1B).

Our study evaluates the seroprevalence of AAs in a comprehensive screening across large, well-controlled DCA cohorts, including both “within-ataxia control groups” (MSA-C, genetically determined DCA subjects) as well as healthy controls (HC) and another disease control cohort (PD).

Seropositivity was low-prevalent in our DCA cohort (2.2%), which was not significantly higher compared to healthy controls (0%; *p* = 0.111) or to PD patients (0.9%; *p* = 0.382). Also compared to the prevalence of AAs in disease control groups of various neurologic and psychiatric central nervous system (CNS) disorders (1.5%) reported by a systematic literature review (1.5%) [22], the prevalence in the DCA cohort (2.2%) was also not significantly higher.

Seroprevalence of AAs was also not higher in etiologically unexplained DCA subjects (i. e. SAOA subjects) compared to etiologically explained DCA subjects (i. e. MSA-C and genetic ataxia). This suggests that seropositivity for AAs associated with ataxia or targeting cerebellar antigens – including even latest AAs – does not in general explain a relevant portion of etiologically unexplained DCA with their characteristic (i.e. non-acute, slowly progressive) disease course. While results might certainly differ in patients with (sub-) acute onset sporadic ataxia, it is this group of DCA patients that is becoming of novel interest, in particular given the novel concept of LACA [16].

While seropositivity for AAs might not per se explain more etiologically unexplained DCA, it might still impact the clinical disease course of DCA. Cerebellar ataxias triggered by AAs typically show (sub-) acute onset dynamics, more rapid and severe disease progression than in ataxias without autoimmune background – in part similar to the respective clinical characteristics of MSA-C [18]. However, the seropositive DCA group did not show higher disease severity or higher CSPR than the seronegative DCA group, indicating that seropositivity for cerebellar AAs does also not generally act as an aggravating disease-modifying factor in DCAs.

These findings on the group level do, of course, not exclude the possibility that pathogenic AAs might still modify clinical characteristics in single seropositive DCA patients, e. g. by accelerated disease progression. Compatible with this notion, one GA patient positive for Recoverin antibodies had a remarkably higher CSPR (4.8 vs 1.3 ± 1.1 points in the SARA score/year in the seropositive GA group). While this might potentially indicate that AAs could contribute to disease progression in single cases, such a single-case finding is speculative in nature and needs to be corroborated by larger group data of AA-seropositive DCA patients. Yet, this case highlights that diagnostic screening for AAs should certainly be initiated in DCA patients with clinical red flags [17], in particular unusual rapid disease progression. However, results of routine CSF analyses (CSF white cell count, CSF total protein, CSF lactate, albumin CSF/serum quotient, CSF immunoglobulin G index) available in 5 of the 6 seropositives – including the recoverin positive case – all yielded unremarkable findings. Still larger DCA cohorts are warranted to systematically identify and analyze single-antibody positive cases in an aggregated fashion.

Another crucial point in the evaluation of AAs is the confirmation by a second, independent laboratory method, e. g. neuronal tissue IFA. In this study, four out of six seropositive samples (SAOA n = 1; GA n = 2; PD n = 1) showed a corresponding reaction in neuronal tissue IFA. In two of the six samples (SAOA n = 1; GA n = 1) the negative findings might be explained by very low titers of antibodies in cell-based assays. In a hypothetical approach where neuronal tissue IFA is the first screening step, these two cases would have been assessed as negative.

Our study has several limitations. First, the sample size of the seropositive group consisting of 6 individuals was small, which limited the power of our statistical analysis of clinical characteristics. Second, results of CSF analyses were only available for 5 of the 6 seropositive individuals based on available medical records, but not for AA testing anymore. Therefore, we were not able to correlate antibody positivity in serum and CSF as it is recommended to screen for AAs in both matrix types to evaluate AA pathogenicity.

Taken together, we found low seroprevalence and low-titre seropositivity for AAs targeting cerebellar antigens in sporadic DCA – similar as in MSA-C, genetically explained DCAs and other neurodegenerative disease controls. This suggests that AAs do not overall substantially contribute to DCAs. Nonetheless, seropositivity for AAs might be pathophysiologically relevant in individual cases modifying clinical characteristics depending on antigen target and antibody titre.

While our study presents the already largest and best controlled study of AAs in DCA – including even latest AAs and “within-ataxia” control groups –, validation of our findings in studies with even larger cohorts including sporadic, genetic and otherwise explained DCAs is warranted.

### Supplementary Information

Below is the link to the electronic supplementary material.

Supplementary file1 (DOCX 35 KB)

## Data Availability

Data supporting the findings of our study is available upon reasonable request to the corresponding author.

## Abbreviations

DCA: Degenerative Cerebellar Ataxias
PD: Parkinson’s disease
AAs: Autoimmune Antibodies
SAOA: Sporadic Adult-Onset Ataxia
MSA-C: Multiple System Atrophy cerebellar type
GA: Genetic Ataxia
SARA: Scale for the Assessment and Rating of Ataxia
CSPR: Cross-sectional progression rate
