# Supplementary material for: Seroprevalence of autoimmune antibodies in degenerative ataxias: a broad, disease-controlled screening in 456 subjects
Source: J Neurol. 2023 Jul 28;270(11):5649–54. doi: 10.1007/s00415-023-11900-1 (PMC10576697; doi:10.1007/s00415-023-11900-1)
Supplement: Supplementary file 1 — Supplementary file1 (DOCX 35 KB) [file 415_2023_11900_MOESM1_ESM.docx]

**Supplementary Material**

**Supplementary methods: Immunfluorescence Assay**

Indirect immunofluorescence assays (IFA) were conducted using slides with a biochip array of brain tissue cryosections (hippocampus of rat, cerebellum of rat and monkey) combined with recombinant HEK293 cells separately expressing 50 different brain antigens. Each mosaic was incubated with 30 µl of sample diluted in PBS, 0.2% Tween-20 (IFA buffer) at room temperature for 30 min, flushed with IFA buffer and immersed in IFA buffer for 5 min. Subsequently, polyclonal goat anti-human pan-IgG (Euroimmun, Lübeck, Germany) labeled with fluorescein isothiocyanate (FITC), were incubated at room temperature for 30 min. Slides were then washed again, embedded in PBS-buffered, DABCO-containing glycerol (approximately 10 µl per mosaic) and examined by two independent observers using an EUROStar microscope (Euroimmun, Lübeck, Germany). Positive and negative controls were included. Samples were categorized based on tissue patterns and fluorescence intensity of transfected cells in direct comparison with non-transfected cells and control samples. Endpoint titers (see Supplementary table 1 for the respective titers) refer to the highest dilution showing visible fluorescence.

**Supplementary table 1: Autoimmune antibody panel in alphabetical order**

| Autoantibody antigen | Abbreviation | Group | Method | Cut off | Reference |
| --- | --- | --- | --- | --- | --- |
| Adaptor-Related Protein Complex 3 Beta 2 Subunit | AP3B2 | 2 | IFA | 1:10 |  |
| Alpha-amino-3-hydroxy-5-methyl-4-isoxazolepropionic acid receptor subunits 1 / 2 | AMPAR1 / AMPAR2 | 2 | IFA | 1:10 |  |
| Amphiphysin | Amphiphysin | 1 | IFA  E | 1:100  + |  |
| Anti-Neuronal Nuclear Antibody type 1 | ANNA-1 / Hu | 1 | IFA  E | 1:10  + |  |
| ATPase Na+/K+ Transporting Subunit Alpha 3 | AT1A3 | 2 | IFA | 1:10 |  |
| Carbonic Anhydrase Related Protein VIII | CARP VIII | 2 | IFA | 1:10 |  |
| Cell adhesion molecule IgLON family member 5 | IgLON5 | 2 | IFA | 1:10 |  |
| Cerebellar Degeneration Related Protein 2 | CDR2 / Yo | 1 | IFA | 1:100 |  |
| Collapsin Response Mediator Protein 5 | CRMP5 / CV2 | 1 | IFA | 1:10 |  |
| Contactin 1 | Contactin 1 | 2 | IFA | 1:10 |  |
| Delta/Notch-like Epidermal growth factor–Related receptor | DNER | 1 | IFA | 1:10 |  |
| Dipeptidyl-Peptidase-like Protein 6 | DPPX | 2 | IFA | 1:10 |  |
| Dopamine receptor D2 | DRD2 | 2 | IFA | 1:10 |  |
| ELKS/RAB6-Interacting/CAST Family Member 1 | ERC1 | 2 | IFA | 1:10 |  |
| Flotillin | Flotillin | 2 | IFA | 1:10 |  |
| Gamma-Aminobutyric Acid receptor A1 | GABARA1 | 2 | IFA | 1:10 |  |
| Gamma-Aminobutyric Acid receptor B3 | GABARB3 | 2 | IFA | 1:10 |  |
| Glycin Receptor subunit alpha-1 | GLAR1b | 2 | IFA | 1:10 |  |
| Glial Fibrillary Acidic Protein | GFAP | 2 | IFA | 1:100 |  |
| Glutamate receptor D2 | GLURD2 | 2 | IFA | 1:10 |  |
| Homer scaffolding protein 3 | Homer-3 | 2 | IFA | 1:100 |  |
| Inositol 1,4,5-Triphosphate Receptor type 1 | ITPR1 | 2 | IFA | 1:100 |  |
| Metabotropic Glutamate receptor 1 | mGluR1 / GRM1 | 2 | IFA | 1:10 |  |
| Metabotropic Glutamate receptor 5 | mGluR5/ GRM5 | 2 | IFA | 1:10 |  |
| Myelin Oligodendocyte Glycoprotein | MOG | 2 | IFA | 1:10 |  |
| Neurexin | Neurexin | 2 | IFA | 1:100 |  |
| Neuro-oncological Ventral Antigen 1 / Anti-Neuronal Nuclear Antibody type 2 | NOVA1 / ANNA-2 / Ri | 1 | IFA | 1:10 |  |
| Neurochondrin | Neurochondrin | 2 | IFA | 1:100 |  |
| Neurofascin 155 | Neurofascin 155 | 2 | IFA | 1:10 |  |
| Neurofascin 186 | Neurofascin 186 | 2 | IFA | 1:10 |  |
| Paraneoplastic antigen Ma2 | PNMA2 / Ma2 | 1 | IFA  E | 1:10  + |  |
| Potassium voltage-gated channel subfamily A member 2 | KCNA2 | 2 | IFA | 1:10 |  |
| Recoverin | Recoverin | 2 | IFA  E | 1:10  + |  |
| Regulator of G-protein signaling 8 protein | RGS8 | 2 | IFA | 1:10 |  |
| Rho GTPase Activating Protein 26 | ARHGAP26 | 2 | IFA | 1:10 |  |
| Rho-associated Coiled-coil-containing protein Kinase 2 | ROCK2 | 2 | IFA | 1:10 |  |
| Sez6-like protein 2 | Sez6l2 | 2 | IFA | 1:10 |  |
| Zinc finger protein of the Cerebellum 4 | ZIC4 | 1 | IFA | 1:10 |  |

Group 1: Recombinant immunofluorescence test not yet finally validated; EUROLINE as validated reference test; clinical relevance of the autoantibody published.

Group 2: Recombinant immunofluorescence test not yet finally validated; clinical relevance of the autoantibody published.

Methods: IFA = recombinant immunofluorescence assay; E = EUROLINE.

**Supplementary table 2: Overview of diagnoses of the genetic ataxia group**

| Genetic ataxia diagnosis | Gene | n of patients |
| --- | --- | --- |
| Ataxia with ocular apraxia type 2 (AOA2) | *SETX* | 4 |
| Autosomal-Recessive Spastic Ataxia Charlevoix-Saguenay (ARSACS) | *SACS* | 5 |
| Ataxia teleangiectasia (AT) | *ATM* | 2 |
| Charcot–Marie–Tooth disease type 2A (CMT2A) | *MFN2* | 1 |
| Charcot-Marie-Tooth disease type 4B3 (CMT4B3) | *SBF1* | 1 |
| Developmental and Epileptic Encephalopathy-93 (DEE93) | *ATP6VA1* | 1 |
| Episodic Ataxia type 1 | *KCNA1* | 1 |
| Friedreich ataxia | *FXN* | 12 |
| Harel-Yoon syndrome | *ATAD3A* | 1 |
| KCNC1-related disorders | *KCNC1* | 1 |
| Marinesco-Sjögren syndrome (MSS) | *SIL1* | 1 |
| Morbus Alexander | *GFAP* | 2 |
| MT-ATP6-associated disease | *MT-ATP6* | 1 |
| Niemann Pick disease type C | *NPC1* | 1 |
| *PNPLA6*-related disorders | *PNPLA6* | 2 |
| *POL3A*-related disorders | *POLR3A* | 1 |
| Polymerase Gamma related ataxia | *POLG* | 5 |
| Spinocerebellar Ataxia type 1 (SCA1) | *ATX1* | 8 |
| Spinocerebellar Ataxia type 2 (SCA2) | *ATX2* | 4 |
| Spinocerebellar Ataxia type 3 (SCA3) | *ATX3* | 9 |
| Spinocerebellar Ataxia type 5 (SCA5) | *SPTBN2* | 1 |
| Spinocerebellar Ataxia type 6 (SCA6) | *CACNA1A* | 4 |
| Spinocerebellar Ataxia type 7 (SCA7) | *ATXN7* | 1 |
| Spinocerebellar Ataxia type 8 (SCA8) | *ATXN8* | 1 |
| Spinocerebellar Ataxia type 14 (SCA14) | *PRKCG* | 2 |
| Spinocerebellar Ataxia type 15 (SCA15) | *ITPR1* | 2 |
| Spinocerebellar Ataxia type 17 (SCA17) | *TBP* | 2 |
| Spinocerebellar Ataxia type 19/22 (SCA19/22) | *KCND3* | 1 |
| Spinocerebellar Ataxia type 21 (SCA6) | *TMEM240* | 1 |
| Spinocerebellar Ataxia type 28 (SCA28) | *AFG3L2* | 1 |
| Spinocerebellar Ataxia type 48 (SCA48) | *STUB1* | 1 |
| *SYNE1*-related ataxia | *SYNE1* | 4 |
| *TSFM-*related disorder | *TSFM* | 1 |
| Autosomal-Dominant Cerebellar Ataxia (ADCA; i. e. ataxia in two or more generations, but SCA gene not yet identified) | - | 14 |
| Autosomal-Recessive Cerebellar Ataxia (ARCA; i. e. ataxia in two or more siblings in one generation, but not in the parents, but ARCA gene not yet identified) | - | 1 |
| Otherwise genetically related ataxia | *-* | 5 |
| *Total* | | *105* |

**Supplementary table 3:**

| ***Seropositive group*** | | | | | | | |
| --- | --- | --- | --- | --- | --- | --- | --- |
|  | ***Degenerative Cerebellar Ataxias (DCA)*** | | | | ***PD***  ***(n= 1)*** | ***HC***  ***(n= 0)*** | ***p*** |
|  | ***SAOA***  ***(n= 2)*** | ***MSA-C***  ***(n= 0)*** | ***Genetic***  ***(n= 3)*** | ***Overall***  ***(n= 5)*** |  |  |  |
| **Females, % ^a^** | 1 (50.0) | - | 0 (0) | 1 (20.0) | 1 (100.0) | NA | *ns* |
| **Age, years** | 50.5 (14.9) | - | 55.7 (19.4) | 53.6 (15.9) | 70.0 (-) | NA | *ns* |
| **Age at onset, years** | 40.5 (12.0) | - | 39.0 (17.0) | 39.6 (13.5) | NA | NA | *ns* |
| **Disease duration, years** | 10.0 (2.8) | - | 16.7 (4.5) | 14.0 (5.1) | NA | NA | *ns* |
| **SARA score** | 6.3 (4.6) | - | 8.8 (7.4) | 7.8 (5.9) | NA | NA | *ns* |
| **Cross-sectional progression rate** | 0.7 (0.7) | - | 0.6 (0.6) | 0.7 (0.5) | NA | NA | *ns* |
| ***Seronegative group*** | | | | | | | |
|  | ***Degenerative Cerebellar Ataxias (DCA)*** | | | | ***PD***  ***(n= 124)*** | ***HC***  ***(n= 125)*** | ***p*** |
|  | ***SAOA***  ***(n= 69)*** | ***MSA-C***  ***(n= 52)*** | ***Genetic***  ***(n= 102)*** | ***Overall***  ***(n= 223)*** |  |  |  |
| **Females, % ^a^** | 27 (39.1) | 25 (48.1) | 48 (47.1) | 100 (44.8) | 58 (46.8) | 59 (47.2) | *ns* |
| **Age, years** | 61.3 (15.1) | 68.2 (9.6) | 50.7 (16.4) | 58.0 (16.3) | 61.8 (14.2) | 60.8 (14.2) | %% ^b^ \| &&& ^b^ \| §§§ ^b^ |
| **Age at onset, years** | 45.0 (18.1) | 58.6 (9.5) | 29.3 (18.9) | 40.9 (20.6) | NA | NA | %%% ^c^ \| &&& ^c^ \| §§§ ^c^ |
| **Disease duration, years** | 16.3 (10.3) | 10.7 (10.9) | 21.6 (12.1) | 17.4 (12.1) | NA | NA | %% ^c^ \| && ^c^ \| §§§ ^c^ |
| **SARA score** | 14.1 (7.1) | 19.2 (6.8) | 17.2 (7.9) | 16.7 (7.7) | NA | NA | %%% ^c^ \| & ^c^ |
| **Cross-sectional progression rate** | 1.1 (0.6) | 2.5 (1.6) | 1.0 (0.5) | 1.3 (1.1) | NA | NA | %%% ^c^ \| §§§ ^c^ |

**Supplementary table 1: Demographic and clinical characteristics of the Degenerative Cerebellar Ataxia (DCA), disease control Parkinson’s Disease (PD) and healthy control (HC) group separately for the seropositive and the seronegative DCA group.** Significant results are represented with one symbol for *p* < 0.05, two symbols for *p* < 0.01, three symbols for *p* < 0.001 and *ns* for *p* > 0.05. # = overall vs PD; $ = overall vs HC; % = SAOA vs MSA-C; & = SAOA vs Genetic; § = MSA-C vs Genetic. NA = not available; MV = missing values.

a: categorical variable: x^2^ test; absolute values (%)

b: normal distributed continuous variable: Student’s t-test; mean (standard deviation)

c: non-normal distributed continuous variable: Kruskal-Wallis test or Mann-Whitney U test as appropriate; median (interquartile range)
